# Influences on catch-up growth using relative versus absolute metrics: evidence from the MAL-ED cohort study

**DOI:** 10.1186/s12889-021-11120-0

**Published:** 2021-06-29

**Authors:** Stephanie A. Richard, Benjamin J. J. McCormick, Laura E. Murray-Kolb, Pascal Bessong, Sanjaya K. Shrestha, Estomih Mduma, Tahmeed Ahmed, Gagandeep Kang, Gwenyth O. Lee, Jessica C. Seidman, Erling Svensen, Margaret N. Kosek, Laura E. Caulfield, MAL-ED Network Investigators, Angel Mendez Acosta, Rosa Rios de Burga, Cesar Banda Chavez, Julian Torres Flores, Maribel Paredes Olotegui, Silvia Rengifo Pinedo, Mery Siguas Salas, Dixner Rengifo Trigoso, Angel Orbe Vasquez, Imran Ahmed, Didar Alam, Asad Ali, Zulfiqar A. Bhutta, Shahida Qureshi, Muneera Rasheed, Sajid Soofi, Ali Turab, Aisha Yousafzai, Anita K. M. Zaidi, Ladaporn Bodhidatta, Geetha Ammu, Sudhir Babji, Anuradha Bose, Ajila T. George, Dinesh Hariraju, M. Steffi Jennifer, Sushil John, Shiny Kaki, Gagandeep Kang, Priyadarshani Karunakaran, Beena Koshy, Robin P. Lazarus, Jayaprakash Muliyi, Preethi Ragasudha, Mohan Venkata Raghava, Sophy Raju, Anup Ramachandran, Rakhi Ramadas, Karthikeyan Ramanujam, Anuradha Rose, Reeba Roshan, Srujan L. Sharma, E. Shanmuga Sundaram, Rahul J. Thomas, William K. Pan, Ramya Ambikapathi, J. Daniel Carreon, Viyada Doan, Christel Hoest, Stacey Knobler, Benjamin J. J. McCormick, Monica McGrath, Mark A. Miller, Stephanie Psaki, Zeba Rasmussen, Stephanie A. Richard, Jessica C. Seidman, Michael Gottlieb, Dennis R. Lang, Karen H. Tountas, Erling Svensen, Caroline Amour, Eliwaza Bayyo, Estomih R. Mduma, Regisiana Mvungi, Rosemary Nshama, John Pascal, Buliga Mujaga Swema, Ladislaus Yarrot, Carl J. Mason, Tahmeed Ahmed, A. M. Shamsir Ahmed, Md Ashraful Alam, Rashidul Haque, Umma Haque, Md Iqbal Hossain, Munirul Islam, Mustafa Mahfuz, Dinesh Mondal, Baitun Nahar, Fahmida Tofail, Ram Krishna Chandyo, Prakash Sunder Shrestha, Rita Shrestha, Manjeswori Ulak, Aubrey Bauck, Robert E. Black, Laura E. Caulfield, William Checkley, Margaret N. Kosek, Gwenyth O. Lee, Kerry Schulze, Pablo Peñataro Yori, Laura E. Murray-Kolb, A. Catharine Ross, Barbara Schaefer, Suzanne Simons, Laura Pendergast, Cláudia B. Abreu, Hilda Costa, Alessandra Di Moura, José Quirino Filho, Alexandre Havt, Álvaro M. Leite, Aldo A. M. Lima, Noélia L. Lima, Ila F. Lima, Bruna L. L. Maciel, Pedro H. Q. S. Medeiros, Milena Moraes, Francisco S. Mota, Reinaldo B. Oriá, Josiane Quetz, Alberto M. Soares, Rosa M. S. Mota, Crystal L. Patil, Pascal Bessong, Cloupas Mahopo, Angelina Maphula, Emanuel Nyathi, Amidou Samie, Leah Barrett, Rebecca Dillingham, Jean Gratz, Richard L. Guerrant, Eric Houpt, William A. Petri, James Platts-Mills, Elizabeth Rogawski, Rebecca Scharf, Elizabeth T. Rogawski, Binob Shrestha, Bishnu Bahadur Rayamajhi, Sanjaya Kumar Shrestha, Tor Strand

**Affiliations:** 1 Fogarty International Center/National Institutes of Health, Bethesda, MD USA; 2 The Pennsylvania State University, University Park, PA USA; 3 University of Venda, Thohoyandou, South Africa; 4 Walter Reed, Armed Forces Research Institute of Medical Sciences (AFRIMS) Research Unit, Nepal, (WARUN), Kathmandu, Nepal; 5 Haydom Lutheran Hospital, Haydom, Manyara Tanzania; 6 icddr,b, Dhaka, Bangladesh; 7 Division of Gastrointestinal Sciences, Christian Medical College, Vellore, Tamil Nadu India; 8 The Johns Hopkins University, 615 North Wolfe Street, Room W2041, Baltimore, MD 21205 USA; 9 Haukeland University Hospital, Bergen, Norway; 10 University of Virginia, Charlottesville, VA USA

**Keywords:** Stunting, Underweight, Catch-up growth, Enteric dysfunction, Permeability

## Abstract

**Background:**

Poor growth in early childhood has been considered irreversible after 2–3 years of age and has been associated with morbidity and mortality over the short-term and with poor economic and cognitive outcomes over the long-term. The MAL-ED cohort study was performed in eight low-income settings with the goal of evaluating relationships between the child’s environment and experience (dietary, illness, and pathogen exposure, among others) and their growth and development. The goal of this analysis is to determine whether there are differences in the factors associated with growth from 24 to 60 months using two different metrics.

**Methods:**

Across six MAL-ED sites, 942 children had anthropometry data at 24 and 60 months, as well as information about socioeconomic status, maternal height, gut permeability (lactulose-mannitol z-score (LMZ)), dietary intake from 9 to 24 months, and micronutrient status. Anthropometric changes were in height- or weight-for-age z-score (HAZ, WAZ), their absolute difference from the growth standard median (HAD (cm), WAD (kg)), as well as recovery from stunting/underweight. Outcomes were modeled using multivariate regression.

**Results:**

At 24 months, almost half of the cohort was stunted (45%) and 21% were underweight. Among those who were stunted at 24 months (*n* = 426), 185 (43%) were no longer stunted at 60 months. Most children increased their HAZ from 24 to 60 months (81%), whereas fewer (33%) had positive changes in their HAD. Linear regression models indicate that girls improved less than boys from 24 to 60 months (HAZ: -0.21 (95% CI -0.27, -0.15); HAD: -0.75 (-1.07, -0.43)). Greater intestinal permeability (higher LMZ) at 0–24 months was associated with lower relative and absolute changes from 24 to 60 months (HAZ: -0.10 (-0.16, -0.04); HAD: -0.47 (-0.73, -0.21)). Maternal height (per 10 cm) was positively associated with changes (HAZ: 0.09 (0.03, 0.15); HAD: 0.45 (0.15, 0.75)). Similar relationships were identified for changes in WAZ and WAD.

**Conclusions:**

The study children demonstrated improved growth from 24 to 60 months of age, but only a subset had positive changes in HAD and WAD. The same environmental factors were associated with growth from 24 to 60 months regardless of metric used (change in HAZ or HAD, or WAZ and WAD).

**Supplementary Information:**

The online version contains supplementary material available at 10.1186/s12889-021-11120-0.

## Background

Poor growth in early childhood is associated with increased risk of morbidity and mortality [1–4], as well as with longer-term negative effects on cognitive development and economic productivity [5–7]. Scientific evidence over decades of research suggested that in early childhood, growth faltering can be reversed and catch-up growth can occur through improved nutrition and other inputs (e.g., reduced disease frequency), but after 2–3 years of age, such inputs are less likely to result in catch-up growth [8, 9]. This research emphasized the need for prevention and control of linear growth faltering and stunting in the first 2 years of life, and this has led to a focus on “the first 1000 days”, recognizing as well the importance of the maternal nutrition environment for child growth and development [10].

The literature documenting the reduced likelihood of catch-up growth after 2–3 years of age led many to consider that children who were stunted at age 2 years would never exhibit catch-up growth and remain stunted at later ages. There are now numerous studies published in which children with height-for-age z-scores (HAZ) < - 2 (i.e., stunted) in early childhood were found to have HAZ > - 2 later in childhood or adolescence [11–15], suggesting that the growth of some children does improve after the age of 2 years. For example, a girl who is approximately 4.5 cm shorter than the median length at 6 months old is the equivalent of - 2 HAZ (2.3rd percentile), but the same absolute difference in cm at 24 months old would equate to being - 1.4 HAZ (8th percentile), hence the appearance of a relative recovery toward the median. Leroy et al. [16] made the observation that because normal child growth is heteroscedastic (i.e., the variability of size (height or weight) increases with age), the absolute difference between the median and standard deviation quantiles increases with age. This means that children may continue to grow poorly but their status will fall more within the limits of the distribution. Leroy et al. [16] pointed out that in terms of the absolute difference in height-for-age from the median (HAD, in cm), improvements in HAZ over time do not necessarily represent a recovery of centimeters foregone.

This contradiction between apparent gains in HAZ concomitant with greater absolute HAD challenges our understanding of the impact of programs or other influences on child growth during the pre-school period. Since the publication by Leroy et al. [16], researchers have compared differences in HAZ and HAD by age to evaluate this phenomenon [17, 18]. Here, we use longitudinal data from the Etiology, Risk Factors, and Interactions of Enteric Infections and Malnutrition and the Consequences for Child Health and Development Project (MAL-ED) to evaluate child growth in terms of height and weight (HAZ, HAD, weight-for-age z-score (WAZ), and weight-for-age difference (WAD)) from 24 to 60 months, and to evaluate factors associated with positive changes in relative versus absolute metrics. We ask the following questions: 1) Is there a difference in the assessment of growth from 24 to 60 months when considered in relative versus absolute terms?, and 2) are there differences in the early life factors associated with growth from 24 to 60 months, depending on which metric is used?

## Methods

The overall goal of the MAL-ED longitudinal multi-site birth cohort study was to evaluate the relationships between the child’s environment and experience (dietary, illness, and pathogen exposure, among others) and growth and cognitive development from birth to 24 months [19]. It was conducted in eight sites; however, two sites were excluded from this analysis, one because of anthropometric data collection irregularities (Naushero Feroze, Pakistan (PKN)) and one because the cohort's prevalence of children with HAZ < - 2 is consistent with the WHO standards (Fortaleza, Brazil (BRF)). The six sites that remained in the analysis were: Dhaka, Bangladesh (BGD); Vellore, India (INV); Bhaktapur, Nepal (NEB); Loreto, Peru (PEL); Venda, South Africa (SAV), and Haydom, Tanzania (TZH). Each site enrolled at least 200 children within 17 days after birth who were born singleton to a mother who was at least 16 years of age, and who weighed at least 1500 g at birth and considered generally healthy. Enrollment began in November 2009, with follow up through February 2014, and through additional funding, a follow-up of these children at 60 months was completed in February 2017 [20]. The protocols were reviewed by appropriate Institutional Review Boards (IRB) in each site and written consent was obtained from the family, both for the initial protocol, and for the follow-up. More detailed descriptions of the study protocol have been published [21–24]; here we provide details most relevant for these analyses.

### Anthropometry

Trained field workers visited the households monthly to measure child length and weight during the first 24 months of life and at least quarterly thereafter until 60 months [20], although due to funding and IRB approval gaps, this was inconsistently practiced at the sites. The length (≤ 24 months) or height (> 24 months), hereafter referred to as height, and weight measures were then converted to sex- and age-specific HAZ and WAZ using the WHO 2006 growth standards [25]. Absolute HAD and WAD were calculated by subtracting the WHO reference median height or weight for a child of the same age and sex from the measured value. Changes in HAZ, HAD, WAZ and WAD were calculated by subtracting the 24-month value from the 60-month value. Field workers additionally measured maternal height 2 months after the child was enrolled in the study. For analyses, children were required to have height and weight values at 0, 24, and 60 months of age, allowing for a ± 30-day window. Stunting is defined as HAZ < -2 and underweight is defined as WAZ < -2.

### Socioeconomic status

Household socioeconomic information was collected every 6 months. In order to have a common measure of socioeconomic status across the sites, an indicator was developed combining information on household Water and sanitation status, Assets, Maternal education, and Income (WAMI) [26]. The WAMI index ranges from 0 to 1, with a higher value indicating a higher socioeconomic status for the household. We calculated the mean WAMI score when the child was 30 and 36 months old in order to best represent their socioeconomic status between 24 and 60 months.

### Illness surveillance

Trained fieldworkers visited the homes bi-weekly during the first 24 months of life to query caregivers about signs and symptoms of morbidity for common illnesses [21]. From this, the prevalence and incidence of diarrhea and respiratory illnesses were calculated. Stool samples were taken during each diarrheal episode and tested to determine etiology [27]. Monthly surveillance stools were also collected and subjected to testing to evaluate pathogen carriage.

### Gut function

The lactulose:mannitol (L:M) test [23] was performed at 3, 6, 9, and 15 months to assess the permeability and absorptive capacity of the gut during the first 24 months of life. We generated age and sex standardized z-scores for the L:M ratios [28] (LMZ) and calculated the mean value of these over the first 24 months of life for each child. A greater value indicates greater enteric dysfunction, using the BRF site as the reference. From each of the monthly (non-diarrheal) surveillance stools, three indicators of gut inflammation and permeability were also assessed: neopterin, myeloperoxidase, and alpha-1-antitrypsin [23].

### Diet

From enrollment to 24 months, during the bi-weekly morbidity surveillance, caregivers were queried about breastfeeding and the feeding of other liquids and solids. From 9 to 24 months, trained field workers utilized a quantitative 24-h recall questionnaire to quantify intakes of non-breast milk foods on a monthly basis [22]. The food intakes were transformed into energy, macro-, and micro-nutrients using study-created food composition databases. Due to co-linearity observed in the dietary data components, only energy intake and the protein density of the diet were included in our analysis. Using the residual method [29], we performed a regression of mean protein intake on mean energy intake and considered the residuals as an indicator of usual protein density. A similar process was used for the intakes of micronutrients included in the supplementary materials in order to derive other measures of the nutrient density in the diet.

### Micronutrient status

Blood samples were taken by venipuncture at 7, 15, and 24 months of age to characterize iron, zinc, and vitamin A status of the child. Plasma concentrations of retinol and zinc were used to characterize vitamin A and zinc status, respectively. Plasma ferritin and plasma transferrin receptor (TfR) were assessed as indicators of iron status. At each time point, and at 60 months, hemoglobin concentration was obtained using the HemoCue method to detect anemia. Biochemical concentrations were adjusted for inflammation using plasma alpha-1-acid glycoprotein (AGP) [30], and transformed using a square root function.

### Statistical methods

The distributions of HAZ, HAD, WAZ, and WAD were plotted at 0, 24, and 60 months of age. The relationship between changes in HAZ and HAD (and WAZ and WAD) between 24 and 60 months were plotted and quantified with correlation coefficients (*r*). Children were identified as stunted or underweight at 24 and 60 months and these categories were used to identify the persistence of or recovery from stunting and underweight status. We used linear regression, controlling for study site, to evaluate factors associated with changes in in HAZ, HAD, WAZ, and WAD. Based on previous analyses of factors related to growth [20, 31, 32], socioeconomic status (WAMI), child’s sex, study site, maternal height, and the value of HAZ, HAD, WAZ or WAD at 24 months were included in each base model. We used biologic rationale, data completeness, and stepwise selection (forward and backward improvements to the Akaike Information Criterion (AIC)) to identify other variables for inclusion, which were tested in a model that included all variables in the base model, plus the variable under consideration. A list of the variables that were considered can be found in the supplemental materials (Supplemental Table 1). Models were run in R 3.4.3 (Foundation for Statistical Computing, Vienna, Austria).

## Results

**Fig. 1:**
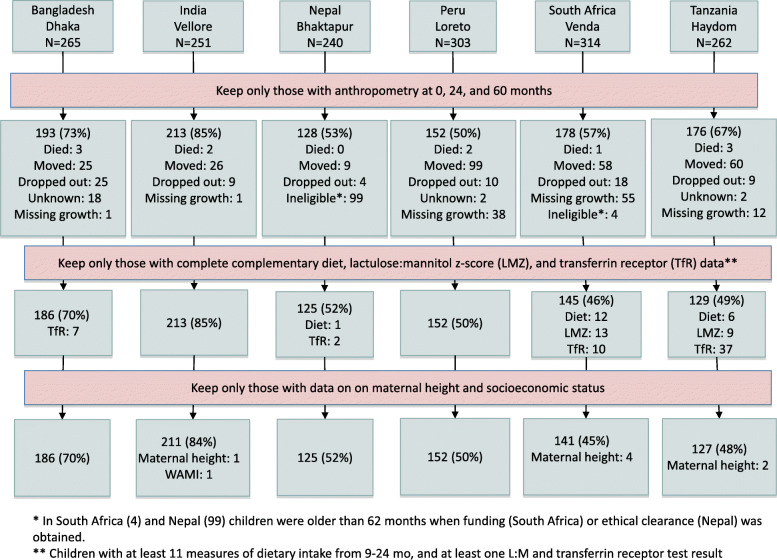
Flow chart of participants included in the analysis from the MAL-ED study

Among the 1635 children enrolled in the six MAL-ED sites included in these analyses, 1040 (64%) had anthropometry at 0, 24, and 60 months of age, and the sample size decreased to 942 (58%) when we required that the children have at least one observation for each of key variables found to be associated with growth outcomes at 60 months in a previous publication using data from this cohort [20]: transferrin receptor, LMZ, maternal height, WAMI, and dietary intake 9–24 months (Fig. 1). We compared the baseline HAZ, WAZ, and WAMI scores between those who were included in the analysis and those who were not and found no statistically significant differences for LAZ at enrollment and mean WAMI scores, but the WAZ at enrollment were statistically significantly lower for those included in this analysis than in those who were not (Supplemental Table 2).

**Fig. 2:**
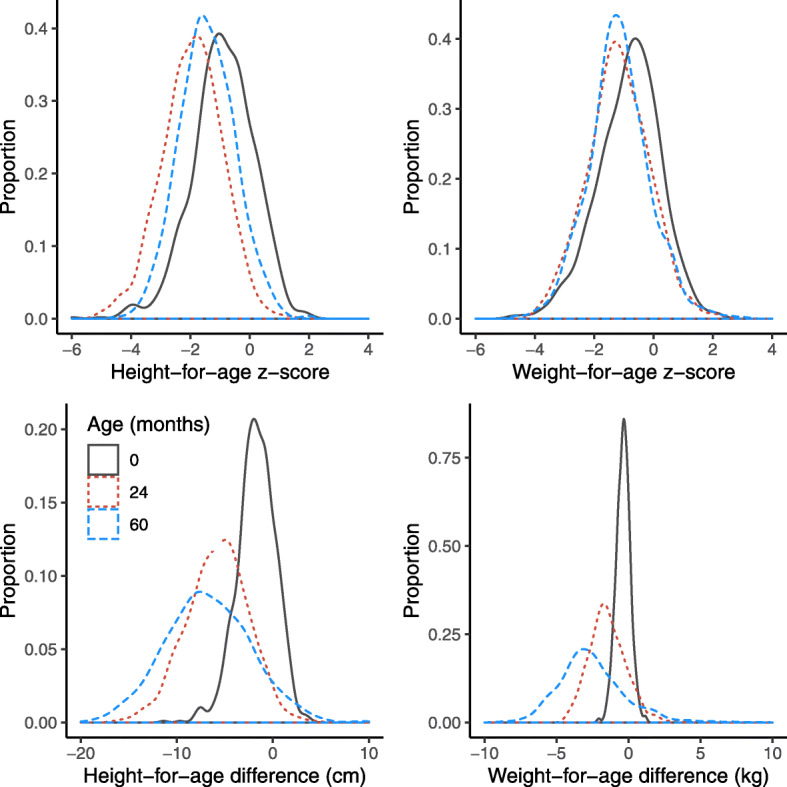
Probability distributions of height-for-age z-scores (HAZ), weight-for-age z-scores (WAZ), and weight-for-height z-scores (WHZ) at 0, 24, and 60 months in the MAL-ED cohort study sites

HAZ values were highest at enrollment, lowest at 24 months, and intermediate at 60 months (Fig. 2 and Table 1). WAZ values were also highest at enrollment, but the values at 24 and 60 months were overlapping. HAD and WAD values were smallest (closer to median) at enrollment and became larger at 24 and at 60 months.

**Table 1:** Characteristics of children with anthropometry at 0, 24, and 60 months of age in the MAL-ED study, including height-for-age z-score (HAZ), height-for-age difference (HAD), weight-for-age z-score (WAZ), and weight-for-age difference (WAD). Stunting is defined as HAZ < -2, underweight is defined as WAZ < -2

N	942
No. girls (%)	474 (50.3)
Mean SES score^a^ (SD)	0.6 (0.2)
Mean Lactulose:Mannitol z-score^b^ (SD)	0.4 (0.6)
Maternal height, cm (SD)	152.2 (6.5)
Mean energy intake^c^, kcal/d (SD)	838.0 (360.8)
Mean protein intake^c^, g/d (SD)	23.3 (10.5)
Mean transferrin receptor^d^, mg/L (SD)	6.0 (3.1)
# stunted at 0 mo (%)	130 (13.8)
# stunted at 24 mo (%)	426 (45.2)
# stunted at 60 mo (%)	256 (27.2)
Mean HAZ at 0 mo (SD)	-0.9 (1.0)
Mean HAZ at 24 mo (SD)	-1.9 (1.0)
Mean HAZ at 60 mo (SD)	-1.4 (0.9)
Change HAZ 24 to 60 mo (SD)	0.5 (0.6)
Change HAD 24 to 60 mo (cm)	-1.0 (2.5)
# underweight at 0 mo (%)	130 (13.8)
# underweight at 24 mo (%)	202 (21.4)
# underweight at 60 mo (%)	184 (19.5)
Mean WAZ at 0 mo (SD)	-0.8 (1.1)
Mean WAZ at 24 mo (SD)	-1.2 (1.0)
Mean WAZ at 60 mo (SD)	-1.2 (1.0)
Change WAZ 24 to 60 mo (SD)	0.0 (0.7)
Change WAD 24 to 60 mo (kg)	-1.1 (1.6)

^a^Socioeconomic score is the mean of values at 30 and 36 months. The score was generated for the MAL-ED study and based on Water and sanitation, Assets, Maternal education, and Income (WAMI) [26]

^b^Lactulose:Mannitol tests were conducted 3, 6, 9 and 15 months and transformed into z-scores, and the mean of 1–4 z-scores was calculated per child

^c^Represents the mean intake per child of non-breast milk foods assessed monthly from 9 to 24 months (range 11–17 assessments per child)

^d^Mean of 1–3 measures per child assessed at 7, 15, and 24 months

Just under half of the children included in this analysis were stunted at 24 months (Table 2), and approximately one-fifth of the children were underweight at 24 months. Children who were not stunted or underweight at 24 months were unlikely to be stunted or be underweight at 60 months (Table 3). Category switches from stunted to non-stunted (and underweight to non-underweight) were common between 24 and 60 months.

**Table 2:** Summary of transitions between stunted and non-stunted status, and underweight and non-underweight status at 24 and 60 months in the cohort. Number (%) of children with positive changes in growth (linear and ponderal), defined as positive change in height-for-age z-score (HAZ), weight-for-age z-score (WAZ), height-for-age difference (HAD), or weight-for-age difference (WAD), and recovery from stunting (HAZ < -2) or underweight (WAZ < -2). Changes were calculated by subtracting the value at 24 months from the value at 60 months of age. Stunting is defined as height-for-age z-score (HAZ) < - 2, underweight is defined as weight-for-age z-score (WAZ) < - 2

Height	N (%)	Weight	N (%)
Not stunted at 24 or 60 months	501 (53)	Not underweight at 24 or 60 months	689 (73)
Not stunted at 24, stunted at 60 months	15 (2)	Not underweight at 24, underweight at 60 months	51 (5)
Stunted at 24, not at 60 months	185 (20)	Underweight at 24, not at 60 months	69 (7)
Stunted at both 24 and 60 months	241 (26)	Underweight at both 24 and 60 months	133 (14)
Positive change in HAZ	763 (81)	Positive change in WAZ	469 (50)
Positive change in HAD	308 (33)	Positive change in WAD	166 (18)
Recovery from stunting	185/426 (43)	Recovery from underweight	69/202 (34)

**Table 3:** Results from linear regression models with the outcomes of height-for-age z-score (HAZ), height-for-age difference (HAD), weight-for-age z-score (WAZ), and weight-for-age difference (WAD) change between 24 and 60 months as a function of anthropometry at 24 months and other child and household factors in six sites from the MAL-ED study (BGD, INV, NEB, PEL, SAV, TZH)

	Δ 24 to 60mo (*n* = 942)			
	ΔHAZ (SE)	ΔHAD (SE)	ΔWAZ (SE)	ΔWAD (SE)
HAZ at 24 mo	- 0.25 (0.02)***			
HAD at 24 mo		0.12 (0.03)***		
WAZ at 24 mo			-0.26 (0.02)***	
WAD at 24 mo				0.33 (0.04)***
SES score, 30–36 mo (10% increase)	0.01 (0.01)	0.03 (0.06)	0.04 (0.02)*	0.09 (0.04)*
Boys 0, Girls 1	-0.21 (0.03)***	-0.75 (0.16)***	- 0.16 (0.04)***	-0.48 (0.09)***
Maternal height (per 10 cm)	0.09 (0.03)**	0.45 (0.15)**	0.10 (0.04)*	0.22 (0.09)*
Mean Lactulose:Mannitol z-score	-0.10 (0.03)***	-0.47 (0.13)***	- 0.13 (0.03)***	-0.28 (0.08)***
Mean energy intake 9–24 mo	0.01 (0.02)	0.07 (0.08)	-0.02 (0.02)	-0.02 (0.05)
Protein density 9–24 mo	-0.01 (0.02)	- 0.03 (0.08)	-0.05 (0.02)*	- 0.11 (0.05)*
Mean transferrin receptor, inflammation adjusted [30]	0.00 (0.01)	0.01 (0.03)	0.02 (0.01)*	0.04 (0.02)*

^***^p < 0.001, ^**^p < 0.01, ^*^p < 0.05

**Fig. 3:**
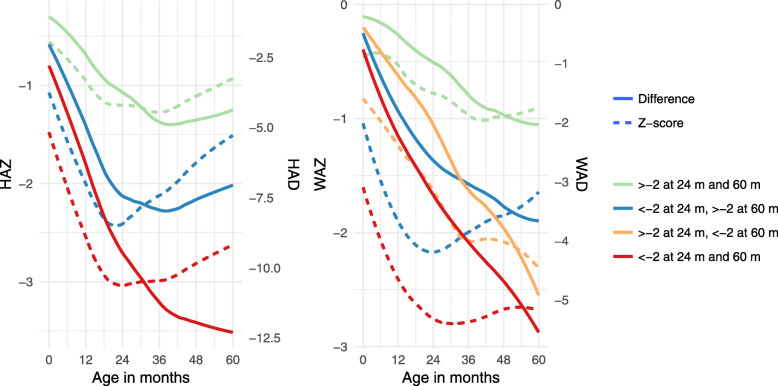
Average z-score (left y-axis, dashed line) and height/weight-for-age difference (right y-axis, solid line) by month of age, among children who were stunted / underweight at 24 and 60 months (red), > - 2 at 24 months and < -2 at 60 months (orange), stunted / underweight at 24 months and not stunted / underweight at 60 months (blue), and not stunted / underweight at either 24 or 60 months (green)

**Fig. 4:**
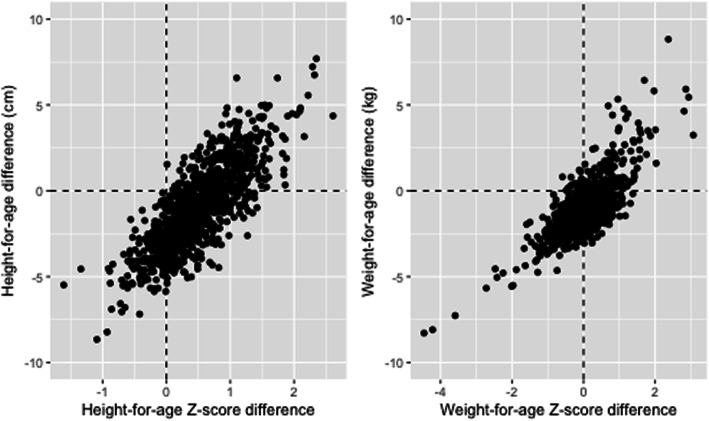
Difference in height/weight-for-age z-scores and height/weight-for-age centimeters/kilograms between 24 and 60 months of age at six MAL-ED sites

Shown in Fig. 3 are the patterns of mean HAZ and HAD amongst those who were never stunted, those who remained stunted and those who recovered from stunting. A figure capturing these same relationships for WAZ and WAD is also shown. Among those who were stunted or underweight throughout, the mean HAD or WAD continued to decrease until nearly 60 months, but flattened out at around 24 months of age for those who were no longer stunted or underweight at 60 months. Changes in HAZ and HAD between 24 and 60 months were highly correlated (*r* = 0.80), as were changes in WAZ and WAD (*r* = 0.77) (Fig. 4).

Most children had a positive change in their HAZ from 24 to 60 months, whereas far fewer had a positive change (smaller difference from median) in their HAD (Table 2). In general, positive changes in WAZ and WAD were less common than for HAZ and HAD.

Results of multivariable linear regression models adjusted for HAZ or HAD at 24 months indicate that child’s sex, maternal height, and mean LMZ were both associated with change in HAZ, HAD, WAZ and WAD from 24 to 60 months (Table 3). Greater average LMZ, a marker of intestinal permeability, was negatively associated with changes in both HAZ and HAD from 24 to 60 months. Results were similar, although not always statistically significant for the Poisson regression models considering positive changes in HAZ or HAD as the outcome variable (Supplemental Table 3). Higher mean TfR in the first 24 months of life was associated positively with changes in WAZ and WAD, whereas the average protein density from complementary foods was negatively associated with changes in WAZ and WAD from 24 to 60 months. WAMI (a measure of socioeconomic status) was positively associated with changes in WAZ and WAD, but when the binary outcomes were considered (positive change in WAZ or WAD, recovery from underweight), the associations with WAMI were not statistically significant (Supplemental Table 3).

## Discussion

Populations with a high prevalence of stunting tend to have height distributions that are negatively shifted, rather than skewed, compared with the standard WHO distribution. This suggests that the entire population, rather than a subset, is not growing to their potential [33]. In these data, from 24 to 60 months of age, the distribution of HAZ shifted to the right, indicating a positive change in height relative to their position at 24 months. However, as has been demonstrated in other studies [16, 18], the HAD distribution shifted further to the left from 24 to 60 months of age, indicating that children had greater absolute height deficits at 60 months than they had at 24 months when compared with the WHO median. For weight, the distributions of WAZ at 24 and 60 months were very similar, whereas the WAD distribution shifted more to the left from 24 to 60 months, indicating greater weight deficits at 60 months.

As discussed earlier, the apparent contradiction between relative and absolute height and weight deficits stems from the increasing variance of normal child growth which is depicted in the WHO z-score calculations. Leroy et al. [16] have suggested that HAD is a more meaningful way to measure catch-up growth in a population, whereas Victora et al. [34] argued that changes in HAZ and HAD are both meaningful ways to express changes in linear growth in children, and that they give complementary information. Here we have shown that in low-income settings, children who show positive changes in HAD are a subset of those who show positive changes in HAZ and are more likely to be those with greater positive changes in HAZ. This is also true for WAZ and WAD, except that positive changes in WAD are much less likely to be observed. Because of the overall high correlation between the changes in the indicators from 24 to 60 months (as shown in Fig. 4), it is not surprising that we did not identify factors in early life that distinguish between these two types of changes over time. Given the similarity of the factors associated with change in HAZ and HAD from 24 to 60 months (child sex, maternal height, and LMZ), both metrics are informative when evaluating environmental influences on the growth of children.

Maternal height was associated positively with change in each of the four metrics from 24 to 60 months. For linear growth (change in HAZ), the association is smaller in effect size but consistent with findings by Addo et al. [35] of positive associations between maternal height and linear growth from 24 months to mid-childhood in five low- and middle-income countries. Here we extend that finding by showing positive associations with change in HAD. As discussed by Addo et al. [35], the persistence of associations between maternal height and linear and somatic growth likely reflect genetic and non-genetic factors as well as intergenerational factors.

Greater average LMZ was associated with negative changes in each of the four outcomes from 24 to 60 months. Greater LMZ is generally regarded as a measure of environmental enteric dysfunction (EED). Previously, we have demonstrated small negative associations with linear growth velocity in the first 24 months of life [36], but statistically significant negative associations with both attained HAZ and WAZ at 60 months [20]. The results here are consistent with this latter finding. Whether EED in early childhood has long-term negative consequences for child growth throughout the pre-school period or whether average LMZ during the first 24 months is a marker for EED from 24 to 60 months is not known.

This hypothesis may also explain findings related to TfR. Previously we have shown positive associations between mean TfR in early childhood and attained WAZ and HAZ at 60 months [20]. Here, the direction of the results is consistent, but the associations are statistically significant for WAZ and WAD only. It is known that TfR is elevated with iron-deficient erythropoiesis and with inflammation (which we adjusted for using AGP), but it is also known to be elevated with cellular proliferation, including erythropoiesis during periods of growth. Thus, associations of TfR in early childhood with growth may reflect greater cellular proliferation, the underlying cause of which is not known. Why variability would be uniquely associated positively with changes in WAZ and WAD from 24 to 60 months requires further research.

That the protein density of complementary foods was negatively associated with changes in WAZ and WAD from 24 to 60 months is somewhat surprising because the same measure was found to be positively associated with growth in weight and length through 24 months [32]. Findings from prior studies (reviewed by Michaelsen and Greer [37]) suggest that higher protein intakes before age 2 years are associated with greater BMI at 4 years of age and older. These findings may not be generalizable to our study children for several reasons. The majority of children in MAL-ED were breastfed through 18+ months of life. Animal milks contributed to the overall protein density of their diet during the first 24 months, but the protein intakes (% energy) were on the order of 10–12%, and the frequency of animal milk consumption declined > 24 months.

One of the primary strengths of this study is the longitudinal data that allow for detailed analysis of growth in early childhood across six sites with high rates of stunting. The extensive data on risk factors collected during the study allow for evaluation of risk factors for growth across different categories, measured in a common way across the sites. However, there are also some limitations to this analysis. Iron deficiency may be underestimated because we adjusted for inflammation using only one marker. There are community-level factors that likely affect the growth of all children at a site, as well as other unmeasured risk factors, and those contribute to the unexplained variance of our models. In addition, the gaps in funding and IRB approvals for the follow up study led to inconsistencies in data collection across sites depending on whether sites could maintain field activities during the gap period and resulted in many children with missing data in some sites. Thus, our analyses necessarily focus on factors assessed during the first 24 months of life, and it may be that longitudinal data sets with more extensive data from 24 to 60 months may be able to identify specific factors during that period associated with positive changes in HAD amongst those with positive changes in HAZ over time. There was considerable loss to follow-up between 2 and 5 years, and this limited our sample size. When we compared some key characteristics, those who were lost to follow up or excluded due to missing variables were similar with respect to mean LAZ at enrollment and socioeconomic status, but had statistically significantly higher WAZ at enrollment than did those who were retained. We expect that the WAZ difference is primarily due to differential follow up at sites (the South African and Peruvian sites had higher WAZ at enrollment than the other sites and higher loss to follow up); because we are controlling for site in the analysis, we expect that to have little effect on our results.

## Conclusions

In six MAL-ED sites, almost half (43%) of the children who were stunted at 24 months of age were no longer stunted at 60 months of age, indicating some degree of improvement in linear growth from 24 to 60 months. Very few children developed stunting after 24 months of age. Most of the children demonstrated some improvement in HAZ, and except for SAV, only about 30% had improvement in HAD. Fewer children had a positive change in WAZ, and most of the children with a positive WAD were in the sites in Peru and South Africa. Given the high correlation and the similarity in factors associated with changes in z-scores or absolute difference, we conclude that both approaches can be used to understand factors associated with child growth from 24 to 60 months.

## Supplementary Information

**Additional file 1.**

## Data Availability

The dataset supporting the analyses presented here can be obtained by requesting the data from the corresponding author. We are not posting this specific file in a repository because data from the MAL-ED study are publicly available (upon request) through the ClinEpiDB platform (https://clinepidb.org). The link to the specific record to explore study data and request a download of the data is https://clinepidb.org/ce/app/record/dataset/DS_5c41b87221

## Abbreviations

BGD: Dhaka, Bangladesh
BRF: Fortaleza, Brazil
HAD: Height-for-age difference
HAZ: Height-for-age z-score
INV: Vellore, India
LAZ: Length-for-age z-score
LMZ: Lactulose:mannitol z-score
NEB: Bhaktapur, Nepal
PEL: Loreto, Peru
PKN: Naushero Feroze, Pakistan
SAV: Venda, South Africa
TfR: Transferrin receptor
TZH: Haydom, Tanzania
WAMI: Water, Assets, Maternal education, and household Income
WAD: Weight-for-age difference
WAZ: Weight-for-age z-score
