# Supplementary material for: Influences on catch-up growth using relative versus absolute metrics: evidence from the MAL-ED cohort study
Source: BMC Public Health. 2021 Jun 29;21:1246. doi: 10.1186/s12889-021-11120-0 (PMC8240385; doi:10.1186/s12889-021-11120-0)
Supplement: Supplementary file 1 — Additional file 1. [file 12889_2021_11120_MOESM1_ESM.docx]

Supplemental Table 1. Linear regression model results from models including listed variable, controlling for site.

| Category | Variable | HAZ | | HAD | | WAZ | | WAD | |
| --- | --- | --- | --- | --- | --- | --- | --- | --- | --- |
|  |  | Beta | P-value | Beta | P-value | Beta | P-value | Beta | P-value |
| Biomarkers | Hemoglobin^1^ | -0.05 | 0.01 | 0.00 | 0.96 | -0.03 | 0.22 | 0.02 | 0.68 |
| Biomarkers | AGP^2^ | 0.00 | 0.01 | 0.00 | 0.22 | 0.00 | 0.38 | 0.00 | 0.70 |
| Biomarkers | Retinol^2^ | -0.01 | 0.13 | 0.00 | 0.95 | 0.00 | 0.84 | 0.00 | 0.63 |
| Biomarkers | MPO^3^ | 0.06 | 0.17 | -0.08 | 0.65 | 0.04 | 0.39 | -0.18 | 0.11 |
| Biomarkers | LMZ^4^ | -0.04 | 0.18 | -0.48 | 0.00 | -0.09 | 0.01 | -0.33 | 0.00 |
| Biomarkers | Zinc^2^ | -0.01 | 0.27 | -0.02 | 0.56 | 0.01 | 0.40 | 0.01 | 0.50 |
| Biomarkers | Alpha-1-Antitrypsin^3^ | -0.04 | 0.37 | -0.53 | 0.01 | 0.06 | 0.26 | -0.15 | 0.25 |
| Biomarkers | Neopterin^3^ | -0.03 | 0.59 | 0.11 | 0.59 | 0.04 | 0.42 | 0.23 | 0.06 |
| Biomarkers | Ferritin^2^ | 0.00 | 0.79 | -0.01 | 0.25 | 0.00 | 0.94 | 0.00 | 0.95 |
| Biomarkers | TFR^2^ | 0.00 | 0.92 | 0.04 | 0.24 | 0.02 | 0.06 | 0.06 | 0.00 |
| Diet 9-24 mo^5^ | Calcium density | -0.06 | 0.00 | -0.10 | 0.23 | -0.05 | 0.03 | -0.04 | 0.46 |
| Diet 9-24 mo^5^ | Zinc density | -0.05 | 0.01 | -0.11 | 0.20 | -0.05 | 0.03 | -0.06 | 0.25 |
| Diet 9-24 mo^5^ | Vitamin A density | -0.04 | 0.03 | -0.08 | 0.33 | -0.01 | 0.74 | -0.01 | 0.89 |
| Diet 9-24 mo^5^ | Animal protein density | -0.04 | 0.04 | -0.04 | 0.60 | -0.04 | 0.10 | -0.02 | 0.70 |
| Diet 9-24 mo^5^ | Vitamin D density | -0.03 | 0.09 | -0.11 | 0.16 | -0.01 | 0.57 | -0.04 | 0.38 |
| Diet 9-24 mo^5^ | Vitamin B12 density | -0.03 | 0.17 | -0.02 | 0.77 | -0.01 | 0.70 | -0.01 | 0.89 |
| Diet 9-24 mo^5^ | Age weaned | 0.00 | 0.27 | 0.00 | 0.73 | 0.00 | 0.27 | 0.00 | 0.14 |
| Diet 9-24 mo^5^ | Protein density | -0.02 | 0.38 | 0.03 | 0.73 | -0.05 | 0.02 | -0.05 | 0.29 |
| Diet 9-24 mo^5^ | Earliest age of predominant BF | 0.00 | 0.44 | 0.00 | 0.28 | 0.00 | 0.83 | 0.00 | 0.88 |
| Diet 9-24 mo^5^ | Minimum age of introduction of milk | 0.00 | 0.50 | 0.00 | 0.14 | 0.00 | 0.09 | 0.00 | 0.22 |
| Diet 9-24 mo^5^ | Maximum age of exclusive BF | 0.00 | 0.57 | 0.00 | 0.47 | 0.00 | 0.72 | 0.00 | 0.05 |
| Diet 9-24 mo^5^ | Vitamin C density | -0.01 | 0.62 | 0.05 | 0.52 | 0.07 | 0.00 | 0.17 | 0.00 |
| Diet 9-24 mo^5^ | Iron density | 0.01 | 0.65 | 0.02 | 0.85 | 0.00 | 0.85 | 0.00 | 0.99 |
| Diet 9-24 mo^5^ | Meat, fish, poultry density | 0.01 | 0.74 | 0.01 | 0.91 | 0.00 | 0.87 | 0.00 | 0.97 |
| Diet 9-24 mo^5^ | Minimum age of introduction of solids | 0.00 | 0.74 | 0.00 | 0.78 | 0.00 | 0.39 | 0.00 | 0.22 |
| Diet 9-24 mo^5^ | Vitamin B6 density | 0.01 | 0.78 | 0.05 | 0.53 | 0.01 | 0.80 | -0.03 | 0.56 |
| Diet 9-24 mo^5^ | Minimum age of partial breastfeeding | 0.00 | 0.85 | 0.00 | 0.18 | 0.00 | 0.89 | 0.00 | 0.02 |
| Diet 9-24 mo^5^ | Energy intake | 0.00 | 0.86 | 0.15 | 0.06 | -0.03 | 0.16 | 0.08 | 0.11 |
| Illness^6^ | ALRI prevalence | 0.00 | 0.11 | 0.00 | 0.68 | 0.00 | 0.63 | 0.00 | 0.68 |
| Illness^6^ | Cough prevalence | 0.00 | 0.11 | 0.00 | 0.20 | 0.00 | 0.05 | 0.00 | 0.02 |
| Illness^6^ | Any hospitalization | 0.10 | 0.29 | 0.45 | 0.28 | 0.08 | 0.50 | 0.23 | 0.39 |
| Illness^6^ | Fever prevalence | 0.00 | 0.51 | 0.00 | 0.87 | 0.00 | 0.12 | 0.00 | 0.30 |
| Illness^6^ | Diarrhea prevalence | 0.00 | 0.53 | 0.00 | 0.31 | 0.00 | 0.31 | 0.01 | 0.06 |
| Illness^6^ | Diarrhea incidence | 0.00 | 0.53 | 0.02 | 0.47 | 0.00 | 0.58 | 0.02 | 0.06 |
| Illness^6^ | ALRI incidence | 0.01 | 0.57 | -0.01 | 0.75 | -0.01 | 0.44 | -0.03 | 0.36 |
| Illness^6^ | Illness prevalence | -0.01 | 0.63 | 0.06 | 0.30 | 0.02 | 0.12 | 0.07 | 0.06 |
| Maternal characteristics | Income | 0.00 | 0.03 | 0.00 | 0.41 | 0.00 | 0.83 | 0.00 | 0.00 |
| Maternal characteristics | WAMI 3-5 y | -0.03 | 0.05 | 0.17 | 0.00 | 0.01 | 0.64 | 0.18 | 0.00 |
| Maternal characteristics | WAMI 0-2 y | -0.03 | 0.06 | 0.16 | 0.01 | 0.01 | 0.57 | 0.18 | 0.00 |
| Maternal characteristics | WAMI 5 y | -0.02 | 0.19 | 0.19 | 0.00 | 0.01 | 0.36 | 0.17 | 0.00 |
| Maternal characteristics | Maternal height | 0.00 | 0.33 | 0.07 | 0.00 | 0.00 | 0.84 | 0.04 | 0.00 |
| Maternal characteristics | Maternal education | 0.00 | 0.96 | 0.07 | 0.01 | 0.01 | 0.24 | 0.07 | 0.00 |
| Pathogens^7^ | *Campylobacter* | 0.03 | 0.01 | 0.01 | 0.79 | 0.02 | 0.14 | -0.04 | 0.14 |
| Pathogens^7^ | Pathogen density | 0.01 | 0.01 | -0.01 | 0.78 | 0.00 | 0.93 | -0.03 | 0.02 |
| Pathogens^7^ | Bacterial density | 0.02 | 0.03 | -0.06 | 0.26 | 0.01 | 0.58 | -0.08 | 0.02 |
| Pathogens^7^ | Pathogen density in first 6 mo | 0.01 | 0.04 | 0.02 | 0.39 | 0.00 | 0.86 | -0.01 | 0.25 |
| Pathogens^7^ | Bacterial density in first 6 mo | 0.01 | 0.06 | 0.01 | 0.77 | 0.01 | 0.42 | -0.02 | 0.42 |
| Pathogens^7^ | ST_ETEC | -0.07 | 0.14 | -0.39 | 0.06 | 0.00 | 0.99 | -0.05 | 0.68 |
| Pathogens^7^ | *Cryptosporidium* | 0.05 | 0.15 | 0.13 | 0.41 | 0.03 | 0.50 | 0.03 | 0.81 |
| Pathogens^7^ | *Giardia* | 0.02 | 0.15 | 0.03 | 0.61 | -0.02 | 0.37 | -0.06 | 0.09 |
| Pathogens^7^ | Viruses | 0.04 | 0.18 | 0.03 | 0.79 | 0.02 | 0.54 | 0.00 | 0.97 |
| Pathogens^7^ | % stools with parasites (per 10%) | -0.01 | 0.19 | -0.06 | 0.01 | -0.02 | 0.00 | -0.06 | 0.00 |
| Pathogens^7^ | ETEC in first 6 mo | -0.01 | 0.47 | -0.11 | 0.19 | -0.01 | 0.64 | -0.04 | 0.43 |
| Pathogens^7^ | Viruses in first 6 mo | -0.01 | 0.48 | -0.09 | 0.24 | -0.02 | 0.39 | -0.06 | 0.18 |
| Pathogens^7^ | EAEC | 0.01 | 0.52 | -0.05 | 0.42 | -0.01 | 0.40 | -0.06 | 0.12 |
| Pathogens^7^ | ETEC | 0.00 | 0.90 | -0.09 | 0.43 | 0.01 | 0.86 | -0.02 | 0.83 |

1 mean of measures from 7, 15, 24 and 60 months

2 mean of measurements at 7, 15, and 24 months

3 mean of log-transformed measurements monthly (1-11 months) and quarterly (12-24 months)

4 mean of age- and sex-standardized ratios from 3, 6, 9 and 15 months

5 mean of the residuals having regressed against total energy

6 prevalence of reported symptoms from 0 to 24 months

7 pathogens detected in monthly stools from 0 to 24 months

Supplemental Table 2. Comparison between those children included and excluded from the analysis. T-tests were used to calculate p-values for difference in means between the two groups.

|  | Included | Excluded |  |
| --- | --- | --- | --- |
| N | 942 | 693 | p-value |
| Mean LAZ at enrolment | -0.92 | -0.95 | 0.54 |
| Mean WAZ at enrolment | -0.84 | -0.69 | <0.01 |
| Mean SES (148 missing any WAMI) | 0.54 | 0.55 | 0.39 |

Supplemental Table 3. Relative risk (95% CI) of positive change in height-for-age z-score (HAZ), positive change in height-for-age difference (HAD), recovery from stunting, positive change in weight-for-age z-score (WAZ), and positive change in weight-for-age difference (WAD) change between 24 and 60 months as a function of anthropometry at 24 months and other child and household factors in six sites from the MAL-ED study (BGD, INV, NEB, PEL, SAV, TZH).

|  | Positive change in HAZ | Positive change in HAD | Recovery from stunting | Positive change in WAZ | Positive change in WAD | Recovery from underweight |
| --- | --- | --- | --- | --- | --- | --- |
|  | (n = 942) | (n = 942) | (n = 426) | (n = 942) | (n = 942) | (n = 202) |
| HAZ at 24 mo | 0.88*  (0.85, 0.91) |  | 3.55*  (2.73, 4.67) |  |  |  |
| HAD at 24 mo |  | 1.07*  (1.03, 1.1) |  |  |  |  |
| WAZ at 24 mo |  |  |  | 0.69*  (0.64, 0.74) |  | 5.95*  (3.27, 11.46) |
| WAD at 24 mo |  |  |  |  | 1.45*  (1.31, 1.61) |  |
| SES score 30-36 mo (10% increase) | 1  (0.97, 1.03) | 1.01  (0.94, 1.09) | 1.07  (0.98, 1.16) | 1.02  (0.97, 1.08) | 1.08  (0.96, 1.21) | 0.98  (0.83, 1.15) |
| Boys 0, Girls 1 | 0.87*  (0.81, 0.92) | 0.69*  (0.57, 0.83) | 0.87  (0.69, 1.09) | 0.8*  (0.7, 0.92) | 0.63*  (0.47, 0.83) | 0.61*  (0.41, 0.89) |
| Maternal height (per 10cm) | 1.05  (0.99, 1.12) | 1.19*  (1.01, 1.42) | 1.14  (0.94, 1.39) | 1.18*  (1.05, 1.34) | 1.04  (0.81, 1.33) | 1.15  (0.79, 1.69) |
| Mean Lactulose:Mannitol z-score | 0.93*  (0.89, 0.98) | 0.89  (0.77, 1.02) | 0.97  (0.83, 1.15) | 0.85*  (0.76, 0.95) | 0.77*  (0.62, 0.96) | 0.53*  (0.35, 0.77) |
| Mean energy intake 9-24 mo | 1  (0.97, 1.03) | 1.05  (0.96, 1.15) | 1.09  (0.97, 1.23) | 0.96  (0.89, 1.02) | 1.04  (0.91, 1.18) | 0.98  (0.79, 1.21) |
| Protein density 9-24 mo | 0.99  (0.96, 1.02) | 1  (0.91, 1.09) | 1  (0.9, 1.11) | 0.94  (0.88, 1.01) | 0.92  (0.8, 1.05) | 0.95  (0.79, 1.14) |
| Mean transferrin receptor | 1  (0.99, 1.01) | 1  (0.97, 1.04) | 0.99  (0.94, 1.04) | 1  (0.98, 1.03) | 1.02  (0.97, 1.07) | 1.04  (0.97, 1.1) |

*p < 0.05
